# Early life predictors of intelligence in young adulthood and middle age

**DOI:** 10.1371/journal.pone.0228144

**Published:** 2020-01-28

**Authors:** Trine Flensborg-Madsen, Hanne-Lise Falgreen Eriksen, Erik Lykke Mortensen

**Affiliations:** 1 Unit of Medical Psychology, Department of Public Health, University of Copenhagen, Copenhagen, Denmark; 2 Center for Healthy Aging, University of Copenhagen, Copenhagen, Denmark; Centre Hospitalier Universitaire Vaudois, FRANCE

## Abstract

**Background:**

Studies on early predictors of intelligence often focus on single or few predictors and often on childhood intelligence. This study compared the contributions of a broad selection of potential early predictors of intelligence at different adult ages.

**Methods:**

Information on predictors was recorded prospectively in the Copenhagen Perinatal Cohort during pregnancy, at delivery, and at 1- and 3-year examinations for children born between 1959–61. Adult intelligence was assessed at three independent follow-ups using three different tests of intelligence: Børge Priens Prøve, Wechsler Adult Intelligence Scale, and Intelligenz-Struktur-Test 2000R. From a total of 4697 cohort members, three non-overlapping samples were derived.

**Results:**

The included predictors explained between 22.2–24.3% of the variance in adult IQ, with parental socioeconomic status and sex explaining 16.2–17.0%. Other consistent predictors were head circumference at birth, increase in head circumference head during the first three years, and 3-year milestones. Head circumference was the most important anthropometric measure compared to measures of weight and length.

**Conclusion:**

Besides social status and sex, the strongest and most consistent early predictors of adult intelligence were physical or behavioural characteristics that to some extent reflect brain–and cognitive development.

## Introduction

Psychometric intelligence or IQ is a major predictor of a broad range of life outcomes such as educational attainment and job performance [1,2] in addition to health outcomes such as morbidity and mortality [3–5]. Identifying early life determinants of adult intelligence is important, not only for understanding the development of intelligence, but also for interpreting potential mechanisms that might explain associations between intelligence and different life outcomes.

Empirical evidence suggests that especially parental education, parental income, and maternal IQ are important predictors of intelligence. Parental education together with maternal IQ and the child’s sex were found to account for 24% of the variance in IQ at age 5 [6]. Maternal and paternal education together accounted for 19% of the variance in IQ at age 8 [7], while paternal social class at birth explained 9–10% of the variance in IQ at ages 7 to 11 [8]. However, only 7.5% of the variance in IQ at age 14 was explained by family income, parental education and breast feeding [9]. According to Mackintosh, correlations between parents’ socio-economic status and children’s IQ scores are generally between 0.30 to 0.35 [1].

In addition to parental demographic factors and parental intelligence, other characteristics of the family background, including parental age and parity [8,10,11], have been investigated as predictors of intelligence. These factors have, however, been found to be only modestly associated with IQ. Studies related to pregnancy and delivery have often focused on specific samples of children born preterm or with low birth weight [12], but general population samples have also identified significant associations [13]. Postnatal factors of importance for later intelligence have included breastfeeding [14,15], institutional care [16], and physical growth of the child, especially in the first year [17]. Finally, early developmental milestones have been documented in several studies to be associated with adult IQ [18,19]. The levels of significance and strength of these early life factors vary across studies which may reflect study differences in the included predictors and covariates as well differences between study samples. Additionally, the majority of studies on early predictors of intelligence uses measures of IQ in childhood, leaving the long-term predictive value of these predictors uncertain.

The objective of the present study was to conduct a systematic evaluation of a broad selection of both well-established and less well-established predictors of IQ in the Copenhagen Perinatal Cohort (CPC). Prospective data were available on a wide range of potential predictors, and this made it possible to estimate the relative contribution of each individual predictor, while taking into account other potential explanatory variables. Additionally, it was possible to derive three non-overlapping study samples with intelligence assessed at three different adult ages (very young adulthood, young adulthood and midlife) and with three different tests of intelligence. Thus, the study will essentially summarize findings from three different studies into one and give an overview of early predictors of intelligence through a substantial part of the adult lifespan.

## Materials and methods

The study is based on the Copenhagen Perinatal Cohort (CPC), which consists of 9125 children born to 8949 mothers at the Copenhagen University Hospital between October 1959 and December 1961. Admittance was based on area of residence (Copenhagen and surroundings), but some were referred due to obstetrical complications or single mother status. When the cohort was established, data on family background, pregnancy and delivery, postnatal factors, postnatal growth, and developmental milestones were recorded prospectively during pregnancy, at delivery, at a 1-year examination, and at a 3-year examination. Detailed descriptions of this data collection can be found elsewhere [20].

Intelligence scores were available for a total of 4697 individuals with data from at least one of the follow-ups. The sample assessed with the WAIS was smallest while the sample tested with the BPP was largest. Consequently, the final WAIS sample included all eligible cohort members, the final IST-2000R sample included all eligible cohort members who were not in the WAIS sample, and the final BPP sample included all male members of the cohort who were not in the WAIS or IST-2000R sample. Administrators of intelligence tests in adulthood were blind to information from CPC.

### Study samples

**The WAIS sample** consisted of individuals from the CPC who participated in the Prenatal Development Project (PDP) follow-up study [21] conducted in 1982–1994. On the basis of perinatal records, 1575 potential participants were invited to the PDP, and 1155 (73%) completed the Danish version of the original WAIS [22]. Of these, 29 twins were excluded, because data for twin pairs are not statistically independent, and the study sample thus comprised 1126 singletons of whom 568 were men and 558 were women. The mean age at follow-up assessment was 27.7 years (range: 20.4–34.5). The full WAIS, including all 11 subtests, was individually administered by three psychologists. IQ scores were derived from Danish test norms [21]. The mean score was 102.2 (SD = 15.2) with a range of 41–142.

**The IST-2000R sample** comprised individuals from the CPC who participated in the Copenhagen Aging and Midlife Biobank (CAMB) [23] during the period from 2009 to 2011. From the CPC 5282 individuals were invited to participate, and a total of 1698 (32.1%) completed the Intelligenz-Struktur-Test 2000R (I-S-T 2000R) [24] (Translated into Danish by Hogrefe Publishers) as part of the clinical CAMB follow-up. Of these, 314 were excluded as they were included in the WAIS sample and 50 were twins; the final sample thus included 1334 singletons of whom 578 were men and 756 were women. The mean age was 50.0 years (range: 48.5–51.4). The I-S-T 2000R is self-administered and consists of three subtests (sentence completion, verbal analogies, and number series), providing a total score ranging from 0 to 59 [25]. The mean score was 28.7 (SD = 10.0) with a range of 4–56.

**The BPP sample** consisted of men from the CPC who appeared before the draft board. With the exception of individuals suffering from disqualifying diseases and individuals who volunteered for military service at an earlier age, all Danish men are required to appear before the draft board [26]. A total of 3307 men from the CPC completed the Børge Priens Prøve (BPP) as part of the draft board assessment. Of these 485 were included in the WAIS sample and 481 were included in the IST-2000R sample, while 104 were twins; the final sample included 2237 male singletons with a mean age of 19.2 years (range 16.3–26.2). The BPP is a 45-minute group test with four subtests (letter matrices, verbal analogies, number series, and geometric figures) providing a total score ranging from 0 to 78 [27]. The man score was 37.9 (SD = 11.4) with a range of 4–71.

### Predictor variables

The following variables were available in the dataset and were considered possible predictors of intelligence; descriptive information on each variable is found in Table 1.

**Table 1:** Study sample characteristics, Copenhagen Perinatal Cohort and associations with intelligence.

	N	Mean (SD)	Range	r^a^ BPP	r^a^ WAIS	r^a^ IST
**Family background**						
Parental socioeconomic status (1 to 8 point scale)	3854	4.2 (1.9)	1–8	0.41***	0.37***	0.36***
Parity (First born %)	4689	49.5	First/Not first	0.07**	-0.01	0.01
Maternal age (years)	4673	25.7 (6.6)	14–48	0.10***	0.13***	0.15***
Paternal age (years)	4515	29.4 (7.7)	14–71	0.06**	0.08**	0.09**
Maternal height (cm)	4577	163.0 (6.0)	139–195	0.11***	0.17***	0.07*
Maternal BMI (kg/m^2^)	4266	21.8 (2.9)	14.3–47.8	-0.04	-0.07*	0.01
Single mother, prenatally (Yes %)	4680	29.5	Yes/No	0.11***	0.13***	-0.14***
**Pregnancy and delivery**						
Sex (Male %)	4697	72.0	Male/Female	-	0.19***	0.18***
Pregnancy complications (yes %)	4697	46.0	Yes/No	-0.04	-0.09**	-0.06*
Delivery Complications (yes %)	4697	40.0	Yes/no	0.02	-0.03	0.02
Gestational age (weeks)	3852	39.2 (2.4)	27–46.5	0.001	0.01	-0.04
Birth weight (grams)	4622	3257 (585)	1050–5500	0.11***	0.10**	0.10***
Birth length (cm)	4621	51.1 (2.7)	38–60	0.13***	0.10***	0.13***
Head circumference at birth (cm)	4305	34.6 (1.6)	25–41	0.12***	0.10***	0.15***
Mother’s smoking in last trimester (Yes %)	4621	49.7	Yes/No	-0.14***	-0.05	-0.07**
Mother’s attitude towards pregnancy (wanted %)	4526	44.3	Yes/No	0.21***	0.19***	0.14***
**Postnatal influences**						
Breastfeeding (months)	3978	3.1 (2.7)	0.3–12	0.14***	0.17***	0.10***
Mother’s employment (employed at 1-year %)	2898	13.1	Yes/No	0.01	0.05	0.07*
Lived in full-time institution at some point in first year (Yes %)	4541	10.2	Yes/No	-0.08***	-0.09**	-0.04
Daycare institution at some point in the first year (Yes %)	4508	13.7	Yes/No	0.01	-0.05	-.0.03
**0–1 year growth and behavioural development**						
Weight increase, first year (kg)	3363	7.3 (1.3)	3.5–20.6	0.01	0.08*	0.06
Length increase, first year (cm)	3325	25.6 (4.0)	13–50.5	-0.06*	-0.03	-0.03
Head increase, first year (cm)	3081	12.9 (1.8)	5.5–22.7	-0.002	0.07	0.05
1-year milestones mean	3505	-0.06 (0.9)	-3.10–3.95	-0.09***	-0.05	-0.07*
**1–3 year growth and behavioural development**						
Weight increase, age 1–3 (kg)	2111	4.6 (1.4)	-4.1.3–13.1	0.12***	0.10*	0.11*
Length increase, age 1–3 (kg)	2129	20.5 (3.8)	0–45	0.14***	0.10**	0.08
Head increase, age 1–3 (kg)	2137	3.5 (1.2)	0–13.5	0.08*	0.08	0.01
3-year milestones mean	2595	0.00 (0.9)	-3.9–3.6	-0.14***	-0.10**	-0.09*

^a^ Pearson correlations were used to evaluate bivariate associations between each predictor and Overall mean of milestones (point-biserial correlation for binary predictors)

*p<0.05;

** p<0.01;

*** p<0.001

#### Family background

*Parental socioeconomic status* (SES) was assessed at the 1-year examination and was based on the social grouping of the Centre International de l’Enfance [28], in which a 0–5 point scale is used to score A) the occupation of the breadwinner, B) the way in which the breadwinner earns his/her wages (public relief, daily wage, weekly wage, monthly salary and own business or capital; C) the education of the breadwinner; and D) the characteristics of the living accommodation (its size, the number of persons per room, its position, etc.) [29] When the data were computerized, the original 0–20 point scale was converted to a scale ranging from 1 to 8 (with 8 indicating the highest SES). *Parity*, *maternal age*, *paternal age*, *maternal height*, *maternal BMI* and *single mother status prenatally* were obtained from interviews by one physician, A. L. Villumsen [30] who interviewed all the women. Contact was established prior to delivery for about 67% of the women.

#### Pregnancy and delivery

*Sex of the child*, *gestational age*, *birth weight*, *birth length*, and *head circumference at birth* were obtained from the postnatal usual routine at the hospital. This was also the case for information on *pregnancy- and delivery complications* which were coded as binary variables for the present analyses. *Mother’s smoking in last trimester* and her *attitude towards pregnancy* were obtained from interviews with the mother.

#### Postnatal influences

Information on *breastfeeding*, *mother’s employment*, *full-time institutionalization* of the child, and *daycare institution* during the first year were obtained from the 1-year follow-up health examination of the mother and the child.

#### 0–1 year growth and behavioral development

*Weight*, *length*, and *head increase during the first year of life* were calculated based on information from the postnatal and the 1-year follow-up health examinations. The *1-year milestones mean* was calculated as the mean age of attaining 12 developmental milestones related to motor development and recorded prospectively during the first year of life by the mother; detailed descriptions of this data collection and the milestones can be found elsewhere [31].

#### 1–3 year growth and behavioral development

*Weight*, *length*, and *head increase from ages 1–3 years* were calculated based on information from the 1-year and the 3-year follow-up. The *3-year milestones mean* was calculated as the mean age of attaining 20 milestones within the areas of language, walking, eating, dressing, social interaction, and toilet training achieved between ages 1–3 years and retrospectively recalled by the mother at a 3-year examination; detailed descriptions of this data collection and the milestones can be found elsewhere [32].

### Data analysis

To evaluate bivariate associations between each potential predictor and IQ at each follow-up, Pearson correlations were used. The rate of missing data on each potential predictor varied from 0% (sex) to 55.1% (weight increase from ages 1–3 years), and due to missing data, all subsequent analyses were conducted using Full Information Maximum Likelihood (FIML) analyses [33]. In the FIML analyses, we used structural equation modelling facilities of Stata 14 (StataCorp LP, USA) to use all available information on covariates in each particular analysis. Preliminary analyses of the WAIS and IST-2000R samples showed no significant interactions between IQ and any of the included predictors wherefore results are shown for the whole sample and are not stratified by sex.

First, a series of multiple linear regression models were conducted for each of the three IQ outcomes (BPP, WAIS and IST-2000R) (Table 2). For each domain of predictors (‘family background’, ‘pregnancy and delivery’, ‘postnatal influences’, ‘0–1 year growth and milestone development’, and ‘1–3 year growth and milestone development’), the predictive strength of the included variables was evaluated. All models included the two core predictors parental SES and sex of the child because these two variables explained a substantial part of the variance in all three IQ tests. Therefore, they were included to obtain a more realistic picture of the effects of other potential predictors in each domain. To avoid excessive col-linearity with maternal height, maternal BMI was not included in the regression models.

**Table 2:** Linear regression analysis of selected predictor variables and milestone means.

	BPP *β*	WAIS *β*	IST-2000R *β*
**Family background**	**Expl. var = 17.5%**	**Expl. var = 19.1%**	**Expl. var = 16.4%**
	**Increase = 1.2%**	**Increase = 2.1%**	**Increase < 0.001%**
Parental socioeconomic status	0.39***	0.36***	0.34***
Sex (men)	—	0.19***	0.17***
Parity (first born)	0.12***	0.05	0.06*
Maternal age	0.03	0.01	0.06
Paternal age	-0.05^1^	-0.05	-0.06
Maternal height	0.06**	0.11***	0.01
Maternal BMI	-0.01	-0.02	0.03
Single mother, prenatally	-0.05*	-0.01	-0.06^1^
**Pregnancy and delivery**	**Expl. var = 18.2%**	**Expl. var = 18.0%**	**Expl. var = 17.7%**
	**Increase = 2.0%**	**Increase = 1.0%**	**Increase = 1.3%**
Parental socioeconomic status	0.34***	0.33***	0.34***
Sex (men)	-	0.16***	0.13***
Pregnancy complications	-0.03^1^	-0.03	-0.03
Delivery Complication	0.00	-0.03	-0.00
Gestational age	-0.06*	-0.01	-0.10**
Birth weight	-0.09^1^	-0.02	-0.05
Birth length	0.10*	-0.00	0.09
Head circumference at birth	0.11**	0.09^1^	0.12*
Mother’s smoking in last trimester	-0.06**	-0.01	-0.00
Mother’s attitude towards pregnancy (wanted)	0.09***	0.06^1^	-0.00
**Postnatal influences**	**Expl. var = 17.4%**	**Expl. var = 18.3%**	**Expl. var = 17.0%**
	**Increase = 1.2%**	**Increase = 1.4%**	**Increase = 0.7%**
Parental socioeconomic status	0.40***	0.35***	0.36***
Sex (men)	-	0.18***	0.18***
Breastfeeding (months)	0.07***	0.10***	0.06*
Mother’s employment (employed at 1-year)	0.03	0.06	0.07^1^
Lived in full-time institution at some point in first year	-0.02	-0.03	0.02
Daycare institution at some point in the first year	0.05*	-0.01	-0.02
**0–1 year growth and behavioural development**	**Expl. var = 17.8%**	**Expl. var = 18.6%**	**Expl. var = 18.4%**
	**Increase = 1.5%**	**Increase = 1.6%**	**Increase = 2.1%**
Parental socioeconomic status	0.40***	0.36***	0.36***
Sex (men)	-	0.16***	0.16***
Weight increase, first year	0.06	0.11**	0.05
Length increase, first year	-0.09*	-0.11*	-0.06
Head increase, first year	0.05	0.06	0.08*
1-year milestones mean	-0.11***	-0.05	-0.11***
**1–3 year growth and behavioural development**	**Expl. var = 19.3%**	**Expl. var = 19.8%**	**Expl. var = 18.5%**
	**Increase = 3.1%**	**Increase = 2.8%**	**Increase = 2.1%**
Parental socioeconomic status	0.38***	0.35***	0.35***
Sex (men)	-	0.22***	0.20***
Weight increase, age 1–3	0.03	0.05	0.12*
Length increase, age 1–3	0.07^1^	0.04	-0.06
Head increase, age 1–3	0.05^1^	0.03	-0.03
3-year milestones mean	-0.13***	-0.14***	-0.11**

^1^p<0.10;

*p<0.05;

** p<0.01;

*** p<0.001

Second, predictors with a p-value of 0.10 or below for at least one IQ-measure in the analyses of each predictor domain were included in a full regression model that also included the two core predictors (Table 3). This selection criterion was chosen to avoid excluding marginally significant predictors that could potentially gain significance in a model with predictors from other domains. Standardized regression coefficients are shown for the FIML analyses and so is the variance explained by each of the five domains and the full model (in addition to the variance explained by parental SES and sex). Table 4 presents an overview of the increase in variance explained by each model of predictors in addition to the variance explained by parental SES and sex.

**Table 3:** Linear regression analysis of selected predictor variables. (Models include all listed variables).

	BPP *β*	WAIS *β*	IST-2000R *β*
	Expl. var = 23.7%	Expl. var = 24.3%	Expl. var = 22.2%
	Increase = 7.5%	Increase = 7.3%	Increase = 5.9%
**Family background**			
Parental socioeconomic status	0.34***	0.31***	0.31***
Sex (women)	-	0.15***	0.12***
Parity (first born)	0.06*	0.02	0.04
Paternal age	-0.05*	-0.04	0.00
Maternal height	0.01	0.09**	-0.03
Single mother, prenatally	-0.02	-0.03	-0.08*
**Pregnancy and delivery**			
Pregnancy complications	-0.02	-0.02	-0.04
Gestational age	-0.06*	0.00	-0.09*
Birth weight	-0.07	0.01	-0.03
Birth length	0.10*	-0.05	0.08
Head circumference at birth	0.16***	0.14**	0.17**
Mother’s smoking in last trimester	-0.04	-0.00	-0.04
Mother’s attitude towards pregnancy (wanted)	0.09***	0.05	-0.03
**Postnatal influences**			
Breastfeeding	0.04	0.09**	0.02
Mother’s employment (employed at 1-year)	0.04	0.07	0.07
Daycare institution at some point in the first year	0.04	-0.04	-0.02
**0–1 year growth and behavioural development**			
Weight increase, first year	-0.04	0.06	-0.00
Length increase, first year	0.04	-0.09	0.01
Head increase, first year	0.17***	0.18***	0.16**
1-year milestones mean	-0.06*	-0.01	-0.08*
**1–3 year growth and behavioural development**			
Weight increase, age 1–3	-0.04	0.01	0.07
Length increase, age 1–3	0.11*	0.03	-0.03
Head increase, age 1–3	0.11**	0.10*	0.02
3-year milestones mean	-0.11***	-0.12***	-0.10**

*p<0.05;

** p<0.01;

*** p<0.001

**Table 4:** Variance explained (R^2^) by models of predictors.

Statistical model	R^2^		
	BPP	WAIS	IST-2000R
Parental SES and sex	16.2%	17.0%	16.3%
**Increase of explained variance:**			
- Family background	1.2%	2.1%	<0.001%
- Pregnancy and delivery	2.0%	1.0%	1.3%
- Postnatal influences	1.2%	1.4%	0.7%
- 1-year growth and development	1.5%	1.6%	2.1%
- 3-year growth and development	3.1%	2.8%	2.1%
- Final model	7.5%	7.3%	5.9%

Preliminary analyses investigated quadratic terms for all continuous predictors, but found only few significant terms, and the quadratic term was not significant for all three IQ tests for any predictor. Consequently, no quadratic terms were included in the analyses. Analyses of Variance Inflation Factors (VIF) were conducted for the final model. For all three IQ outcomes, the only predictors with VIF>5 were birth weight (5.15–6.44) and birth length (5.14–7.47). According to Danish regulations, the present analyses do not require approval by the scientific ethical committee system.

## Results

### Sample composition

Table 1 shows that the mean maternal and paternal ages were 25.7 and 29.4 years respectively. At least one pregnancy complication was registered for 46% of the study sample and at least one delivery complication was registered for 40%. Almost half of the mothers smoked in the last trimester and 29.5% were single mothers at delivery. A total of 72% of the the study sample were men because BPP scores were only available for men. The mean birth weight was 3257g with a mean weight increase in the first year of 7.3kg. The majority of mothers (86.9%) were not employed in the first year of the child’s life while 13.7% of the children spent time in a daycare institution at some point during the first year.

### Bivariate correlations

Table 1 shows that parental SES and sex had the strongest bivariate correlation with the three IQ-measures. Significant correlations with at least one IQ-measure were observed for 25 of the 29 predictor variables and the following variables showed significant correlations in all three study populations with IQ: Higher maternal age, higher paternal age, larger height of the mother, single mother status, larger birth size (weight, length, and head circumference), mother’s attitude towards pregnancy, longer breastfeeding, higher 3-year weight increase, and faster attainment of milestones in the first three years. In the study sample, the Pearson correlation between WAIS and IST-2000R was 0.79 (n = 314), between WAIS and BPP 0.81 (n = 485), and between IST-2000R and BPP 0.76 (n = 602). The corresponding mean intervals between the assessments were 22.3 years, 8.5 years, and 30.8 years.

### Models of predictor domains

In the models analyzing domains of predictors separately for each of the three IQ-measures, several patterns from the bivariate analyses were repeated (Table 2). Thus, variables from all five domains were significantly associated with IQ, with especially parental SES and sex showing significant associations in all analyses. In the domain of ‘family background’, parity (first pregnancy) was associated with significantly higher BPP and IST-2000R scores while maternal height significantly predicted higher IQ on the BPP and WAIS. In the domain of ‘pregnancy and delivery’, lower gestational age and larger head circumference at birth were significantly associated with higher BPP and IST-2000R scores. In addition, birth length and the pregnancy being wanted positively predicted BPP scores while maternal smoking was negatively associated with BPP scores. Among the ‘postnatal influences’ duration of breastfeeding was positively associated with all three tests of intelligence while daycare institution was a significant positive predictor of BPP scores. Several predictors within the domain of ‘0-1-year growth and behavioral development’ were significant, and length increase was significantly negatively associated with IQ on the BPP and WAIS, while weight increase was positively associated with WAIS IQ and head increase with IST-2000R scores. Low mean age of attaining 1-year milestones significantly predicted higher BPP and IST-2000R scores while low mean age of attaining 1-3-year milestones not only significantly predicted higher scores on all three IQ tests, but in fact showed some the highest standardized regression coefficients. The only significant 1–3 year growth measure was weight increase that positively predicted IST-2000R scores.

### Final model

The mutually adjusted model with selected predictors included variables from the five domain analyses for each of the three IQ-measures with a p value of <0.10 for at least one of the IQ-measures (see Table 3).

Parental SES was the strongest single predictor of intelligence with coefficients between 0.31–0.34 (p<0.001). Sex was significantly associated with both WAIS and IST-2000R (p<0.001), indicating that men scored higher than women. Head circumference at birth in addition to increase in head size during the first year were significantly associated with all IQ-measures with coefficients in the range of 0.14–0.18 while head increase from 1–3 years only significantly predicted BPP and WAIS scores. Another consistent predictor was age of attaining milestones means, with the mean age of milestones at the 3-year follow-up significantly predicting all IQ scores (coefficient range -0.10 –-0.12) and milestones at the 1-year follow-up predicting BPP and IST 2000R scores. Gestational age was negatively associated with both BPP and IST-2000R scores, while the following predictors were significantly associated with only one IQ-measure: Parity, paternal age, maternal height, single mother status, birth length, mother’s attitude towards pregnancy, duration of breastfeeding and length increase from 1–3 years.

### Explained variance

A basic model including only the predictors parental SES and sex explained 16.2%, 17.0% and 16.3% of the variance in BPP, WAIS and IST-2000R respectively. The increase in variance by including predictors in the domains (subtracting the variance of parental SES and sex) ranged between <0.0001% to 2.1%. The largest increase in the amount of variance was generally found for the domain ‘3-year growth and milestone development’, reflecting the strong associations with increase in head size and the 3-year milestone mean.

The final model accounted for 22.1% to 24.3% of the variance in IQ-measures and additional predictors, other than parental SES and sex, together added between 5.9% to 7.5% in explained variance.

## Discussion

We were able to explain between 22.2% and 24.3% of the variance in IQ with a final model summarizing the findings in the five predictor domains. As expected, parental SES was a significant and substantial predictor in all regression models and together with sex, this variable explained 16.2–17.0% of the variance in IQ. Factors other than parental SES and sex explained between 5.9% and 7.5%, with the most consistent predictors being head circumference at birth, head increase at the first and third year follow-ups, and 3-year milestones. Head circumference was the most important size–and growth measure compared to measures of weight and length.

When evaluating the results of this study it is important to consider the role of time for both predictors and outcome follow-ups. Thus, in contrast to many studies of family background and perinatal—and early postnatal predictors, the present study included data on physical and behavioural development during the first three years of life. Thus, the final model shows the direct effect of each variable and not any indirect effects mediated by growth and behavioral development during the first three years of life. This may partly explain why some of the early factors that were significant in bivariate and domain analyses did not remain significant in the final model. Examples could be breastfeeding and weight increase that predict behavioural milestones during the first year of life [31].

The remarkable strength of the association between the broad CPC measure of parental SES and adult intelligence has been discussed previously [34]. This covariation may reflect both genetic and environmental factors [1] and in addition, SES may modify the heritability of intelligence and increase the magnitude of individual differences in intelligence [35]. To some extent, effects of parental SES are also likely to be mediated through environmental factors influencing cognitive development. The relatively small decrease in the strength of the association between parental SES and adult intelligence in the final model suggests that only few of such relevant environmental factors were included in the present study.

Sex was included because sex differences on the original version of the WAIS and the IST-2000R have been reported previously [25,36]. These findings were replicated in this study, but they may not only reflect cognitive differences between men and women, but also test construction factors favoring men. In fact, sex differences in favour of girls were observed for a later generation of Danish five-year old children [6].

The other ‘family background’ variables were not consistently associated with offspring intelligence across the three analysis samples. Maternal and paternal age have previously been associated with offspring cognitive development, but a recent study of ½ million Swedish men found no association between paternal age and offspring intelligence [37] and the weak negative association in this study was only significant in the BPP sample. The positive association of maternal height with offspring intelligence may reflect the known association between height and intelligence, but it is not clear why the effect could only be identified in the WAIS sample. This is also the case for the negative association of single mother status with offspring intelligence observed only in the IST-2000R sample.

The binary measures of pregnancy and delivery complications were not significantly associated with offspring intelligence, but this may reflect inclusion of common conditions such as hypertension and the fact that the study samples consisted of individuals able to participate in the follow-ups or to appear before the draft board.

The predictive validity of anthropometric measures in childhood for IQ has often been reported, and we observed consistently significant bivariate correlations for weight, length and circumference at birth. However, in the final model, only head circumference showed significant associations with all three outcomes, most likely reflecting substantial intercorrelations among the three predictors (range 0.78–0.87). Thus, birth weight has previously been shown to predict intelligence across the adult lifespan in the CPC, but in this study, birth weight was categorized into five categories and the models were not adjusted for birth length and head circumference [13]. Birth weight and length reflect fetal body growth while head circumference may be more specifically related to brain size, and according to Deary et al. [38] correlations between head size and intelligence are about 0.20 while correlations between total brain volume and intelligence are found to be in the range 0.30 to 0.40. In our study, head circumference at birth was among the strongest predictors of offspring intelligence, and this should probably be interpreted in the context of associations between head circumference, brain size and intelligence.

The interpretation is corroborated by the results for the growth variables. In the domain analyses, only length increase during the first year showed a consistently significant association with later IQ, but this pattern was clearly different in the full model where head circumference increase at both the 1-year and 3-year follow-ups showed strong associations with offspring intelligence (except for IST-2000R at the three-year follow-up). Effects of postnatal growth have also been emphasized in other studies where early growth has been linked to intelligence later in childhood [17,39,40] and early head circumference growth may be particularly important for behavioral development [41]. The standardized coefficients suggest that growth in head circumference during the first year may be more important than growth during the subsequent years, and indeed, growth in head circumference during the first year may be among the strongest predictors of adult intelligence.

Significant negative correlations of gestational age with adult intelligence were observed in the BPP and IST-2000R samples. In the full study sample, gestational age and birth weight correlated 0.52 and the negative coefficients may reflect collinearity with the fetal growth measures.

Maternal smoking during pregnancy has previously been found to predict offspring intelligence in the CPC [34]. The smoking variable in that study reflected number of cigarettes smoked while our binary measure of maternal smoking showed a significant association with offspring BPP scores in the domain analyses, but no significant associations in the final model.

Among the postnatal influences on adult intelligence, spending time in a full-time institution showed negative bivariate correlations. However, full-time institution lost significance in the domain analyses, and neither day-time institution nor mother’s employment were significant in the final models. A positive association in the CPC between duration of breastfeeding and offspring intelligence has been demonstrated in a previous study [14], and this association was corroborated by significant associations in the domain analyses, but only for the WAIS sample in the full model. This pattern may to some extent reflect mediation through measures of later growth and behavioral development.

Behavioral development as reflected in mean age of attaining milestones was a quite consistent predictor of adult intelligence, although the age of attaining 1–3 year milestones was a somewhat more consistent and stronger predictor than age of attaining 1-year milestones. This is in accordance with previous findings from CPC suggesting that a substantial part of the effect of 1-year milestones is mediated through 3-year milestones [19]. Behavioral development must to some extent reflect cognitive and brain development, and as the child grows older developmental trajectories tend to become more stable. This may explain why age of attaining 3-year milestones was among the strongest predictors of adult intelligence–an interpretation in line with previous CPC studies showing that especially milestones related to language and social interaction is associated with IQ in adulthood [19,32].

### Methodological considerations

A considerable strength of this study is that intelligence in adulthood was assessed in three different non-overlapping samples with three different measures of intelligence at different adult ages. This allows stronger conclusions concerning the consistent predictors of adult intelligence, but also raises questions concerning the interpretation of different results for the three study samples.

Differences between the predictors identified in the analyses of the three outcomes may to some extent reflect differences between the three measures of intelligence. The BPP and the IST-2000R include similar subtests requiring verbal reasoning while the WAIS is a much more comprehensive measure of intelligence, including 6 verbal—and 5 performance subtests. Nevertheless, the remarkably high intercorrelations among the three intelligence tests despite the long intervals between the follow-up assessments indicate that differences between the three measures probably only play a minor role. Accordingly, differences in results for the three outcomes may also reflect the changing influence of early life factors on intelligence through the adult life-span. Thus, except for SES and sex, 5.9% of the variance was explained by early life factors on the IST-2000R variance while more than 7% was explained for both the BPP and the WAIS. This difference could reflect that the influence of some early life factors are diluted over the life-span, although, some predictors attained the highest coefficients in the IST-2000R analysis (single mother status, head circumference at birth, mean age of attaining 1-year milestones). Finally, there are differences in mean level and variance of many covariates between the three samples and there could be corresponding differences in intelligence means and variance; these differences may also to some extent explain the different findings between the three samples.

For the WAIS sample and IST-2000R samples, participation may be related to intelligence and thus indirectly to some of the predictors included in the study. To the extent that selective participation has reduced the variance in both intelligence and some of the early predictors, the associations may have been diluted in these samples. However, selection bias is not likely to have affected the results for the BPP, although the BPP sample indirectly reflects selection of participants in the PDP and the CAMB follow-ups to the corresponding study samples. The generalizability of this study should also be considered, as the results are based on a Danish birth cohort from 1959–61 characterized by for example a high proportion of pregnancy- and delivery complications (40–46%) in addition to a high proportion of mothers smoking in last trimester (50%) and being unemployed in the first year of the child’s life (87%).

Our analyses are based on one composite score derived from each test of intelligence. Thus, our conclusions concern general intelligence wherefore the study provides no information on prediction of more specific cognitive abilities. However, our results are likely to apply to specific abilities showing high correlations with general intelligence such as verbal reasoning, vocabulary, and spatial ability [38].

A final limitation of the study should be noted. Previous studies have emphasized the importance of maternal IQ as a core predictor of the child’s IQ [6]. Since this information was not available in the present study, it is likely that inclusion of such a measure would significantly increase the amount of variance explained by the predictors and affect levels of significance in both the domain analyses and the final models. Finally, given the potential importance of proximal factors in the first years of life, the lack of a measure describing the home environment, including parent-child interaction, stimulation, and parenting style, is a genuine limitation.

## Conclusion

Taken together, we found in three different and non-overlapping study samples that the early life predictors explained 22.2–24.3% of the variation in adult intelligence. Parental SES (together with offspring sex) was confirmed as a core predictor of IQ, since it was consistently and substantially associated with adult IQ in all three study populations, whereas the other statistically significant predictors only explained a small increase in variance in adult IQ. In addition to emphasizing the importance of including infant SES in studies of early predictors of intelligence, the study also stresses the importance of considering potential residual confounding and indirectly mediated effects when conducting such studies. Thus, while bivariate analyses and some domain analyses replicated findings from previous studies for a variety of significant predictors, several of these were not significant in the final models including variables from all domains (birth weight is an important example). Interpreting studies of early predictors of intelligence should therefore take the included predictors and covariates into account and consider both the measure of intelligence and the timing of assessment of intelligence over the lifespan.

In the final model including the selected predictors from the domain specific analyses, the most consistent predictors were head circumference at birth, head increase in the first year, head increase from age 1 to 3 years, and 3-year milestones. Thus, we conclude that–apart from social status and sex–all the strongest and most consistent early predictors of adult intelligence were physical characteristics (head circumference at birth and growth in head size) and behavioural characteristics (1-year and 3-year behavioural milestones) that to some extent reflect brain- and cognitive development.
